# The effects of 12 weeks of high-intensity interval rope skipping training on speed and power indexes in male soccer players

**DOI:** 10.3389/fbioe.2025.1579535

**Published:** 2025-07-10

**Authors:** Bo Wei, Wenhu Cheng, Jiangang Qiu

**Affiliations:** ^1^ College of Art, Chengdu Sport University, Chengdu, China; ^2^ Track and Field Teaching and Research Department, Chengdu Sport University, Chengdu, China

**Keywords:** randomized controlled trial, jump rope training, sensitivity, speed, power, stretch shortening cycle, soccer

## Abstract

**Research Purpose:**

Speed ability is an important determinant of a soccer player’s competitive performance in a game, and it also affects the athlete’s sports life and upper limit of competitive level. Power is the core of the soccer player physical ability construction and its influence on the speed of permeate, confrontation, technology, endurance and injury prevention and so on each linThe main objective was to investigate physiological adaptations induced by HIIRS on soccer-specific speed and power qualities of soccer players.

**Research Method:**

Twenty-five elite soccer players underwent a 12-week jump rope training program (three 45-min sessions weekly). Participants were randomly assigned to an, EG (n = 13) performing high-intensity intermittent rope skipping (75%–85% HRmax with active intervals at 40%–70% HRmax) and a CG (n = 12) maintaining conventional training (75%–85% HRmax), both monitored via heart rate bands. Pre and post-intervention assessments included: sprint speed (30-m sprint), reaction speed (Optojump 5-s rapid frequency test), visual reaction speed (Optojump visual test), change-of-direction speed (T-test), and explosive power (standing long jump). Data were analyzed via paired samples T-tests.

**Research Results:**

Research Results. The results of the study found that both high-intensity interval rope skipping (HIIRS) training and traditional training significantly improved sprint speed (HIIRS: P < 0.001; Traditional: P = 0.0009), change of direction speed (HIIRS: P = 0.0103; Traditional:P = 0.0130), and explosive speed (HIIRS:P = 0.0315; Traditional: P = 0.0002). Additionally, HIIRS training significantly improved movement speed (P = 0.0405) and visual reaction speed (P = 0.0441), which were not significantly enhanced by traditional training. Further, HIIRS training demonstrated superior effectiveness compared to traditional training specifically forsprint speed (P = 0.0326) and visual reaction speed (P = 0.0101). This study integrates HIIT principles with rope skipping’s biomechanical SSC action to target neuromuscular adaptations.

**Research Conclusion:**

This finding enriches the functionality of the sport of jumping rope and provides an optional training tool for soccer players to develop their speed qualities.

## Introduction

As a plyometric training tool, rope skipping is a quick rebound jumping action, foot, knee and hip flexion is very small, if you pay attention to its muscle action form, then it is a stretch-shortened cycle action (SSC) (Miyaguchi et al., 2014). SSC is to enhance the muscle stiffness of the Muscle-Tendon Complex (MTC) Gastrocnemi -Achilles tendon complex under the function of the extension-reflex mechanism, and realize the combined training of strength output and muscle extension, so as to improve the muscle output power and speed quality. The SSC, characterized by rapid eccentric-concentric muscle transitions, is a fundamental mechanism for enhancing power output and reactive strength (de Villarreal et al., 2011). Jump rope training, inherently reliant on SSC mechanics, has demonstrated efficacy in improving sport-specific attributes such as vertical jump performance in basketball (Ciacci and Bartolomei, 2018) by 18%, punching velocity in boxing (Chottidao et al., 2022) improved 9.2%, and sprint acceleration in track athletes (Miyaguchi et al., 2015) with 5.3% gains. Notably, the rhythmic, multi-planar nature of rope skipping closely mimics the rapid footwork and dynamic balance required in soccer (Trecroci et al., 2015). Despite these advantages, existing studies on jump rope interventions have predominantly focused on isolated performance metrics rather than its integration with HIIT protocols tailored for soccer-specific physiological adaptations (Wu and Huang, 2023; de Villarreal et al., 2011).

Soccer, as a dynamic and intermittent sport, imposes multifaceted physical demands on players, including repeated high-intensity actions such as 10–20 m sprints (Sedano et al., 2009) occurring every 90 s, rapid acceleration-deceleration during dribbling (Ozbar, 2015) with 15–20 directional changes per match, and unpredictable directional changes (Dolci et al., 2021). These speed-related qualities (e.g., sprinting, reactive agility, and explosive power) are critical determinants of match performance and have been directly linked to competitive outcomes (Kabacinski et al., 2022; Morgan et al., 2022). However, traditional soccer training programs often prioritize sport-specific drills (e.g., small-sided games) and linear sprint exercises, which may insufficiently address the neuromuscular adaptations required for multi-directional speed and reactive agility.

While HIIT has been extensively validated for improving aerobic capacity by 12%–15% and anaerobic capacities by 8%–10% in soccer players (Rabbani et al., 2019), its application through non-traditional modalities like jump rope remains underexplored. This study addresses two critical gaps: (1) the lack of evidence on HIIT protocols combining SSC-based jump rope exercises with soccer-specific speed demands, and (2) the need for comparative analyses between innovative and traditional training approaches. We hypothesize that 12 weeks of high-intensity interval rope skipping (HIIRS) training will elicit superior improvements in soccer-specific speed qualities particularly sprint acceleration (>10%), visual reaction time (>15% reduction), and multi-directional agility (>8% improvement) compared to conventional training methods.

Collectively, this study seeks to advance the field of soccer-specific conditioning by:

(1) Establishing HIIRS as a modality synergizing SSC mechanics (biomechanical basis) with HIIT-driven physiological adaptations.
(2) Quantifying its comparative efficacy against conventional methods in enhancing speed qualities.
(3) Providing empirical support for integrating low-cost, versatile tools like jump ropes into elite soccer training regimens.

## Materials and methods

### Ethical approval

This study was approved by the Ethics Committee of Chengdu Physical Education University, with approval number 2023152. The study followed the requirements of the Helsinki Declaration (2013 version) and obtained written informed consent from all participants after fully informing them about the study content. After the participants were fully informed about the study content, they agreed to take part in the study.

### Subjects

This study was a randomized controlled trail (RCT). This study used G-power 3.1.9.7 software to calculate the sample size and analyze the sample size required for the experiment. Analysis conditions: Effect size f = 0.25, α err prob = 0.05, power (1-βerr prob) = 0.8, the required sample size is 10 people in the control group and 10 people in the experimental group, a total of 20 people.

Twenty-five elite male soccer players (age: 19.0 ± 1.5 years) were recruited from Chengdu Sport University and randomly assigned to an experimental group (E.G., n = 13) or control group (CG, n = 12) using computer-generated random numbers (Table 1). Allocation concealment was ensured by sealing assignments in opaque envelopes opened by an independent researcher after baseline assessments. All participants in this study are elite athletes, such as very few athletes, and from nine different provinces in China. Thanks for the expert opinion, this is a writing error, has been revised. The changes are as follows: Recruitment will be completed by 10 March 2024. The experimental intervention was conducted from 15 March 2024 to 20 July 2024. The participants met the following recruitment criteria. Inclusion criteria: ① All participants underwent strict medical examination before recruitment, ensuring good health and the absence of chronic lung disease, cardiovascular disease, and depression. ② All athletes were informed of the precautions and safety requirements of the experiment and signed the informed consent form. ③ They voluntarily participated in this experiment. Exclusion criteria: ① Individuals with chronic diseases who were taking medication. ② Those unable to fully participate in the experimental process. ③ Those unwilling to sign the informed consent form. Withdrawal criteria: ① Subjects in the experimental group who failed to complete more than 90% of the training sessions. ② Subjects who experienced significant discomfort with the training content during the experiment. ③ Subjects who took medication that affected vasoconstriction or vasodilation. The entire process of this experiment and the data collection site were carried out in the indoor track and field stadium of Chengdu Sport University.

**TABLE 1:** Descriptive data of participants (Means ± SD).

Subject information entry	E.G., (N = 15)	CG (N = 115)	P
Age (year)	19 ± 2.5	19.1 ± 1	0.898
Height (cm)	178.23 ± 7.4	177.05 ± 8.5	0.712
Weight (kg)	73.2 ± 8.1	74.6 ± 7.5	0.976
Training periods (year)	9.81 ± 3.5	9.3 ± 2.7	0.881

Note: E.G., experimental group; CG, control group.

### Design

This study employed a single-blind experimental design to minimize bias, where both the subjects and the intervention implementers were blinded to group assignments. Intervention implementers (trainers) were not blinded due to the nature of the training modalities. Specifically, participants were not informed whether they were in the experimental or control group, and the trainers conducting the sessions were unaware of the study’s hypotheses and group comparisons. This blinding was maintained throughout the 12-week intervention period to ensure objective data collection.

**FIGURE 1:**
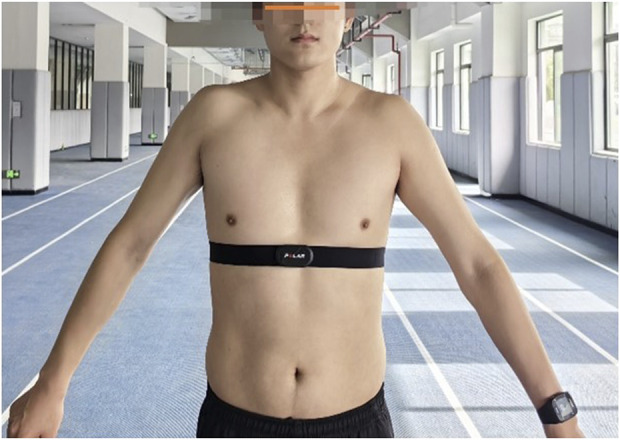
Illustrated guidelines for the proper placement of the polar heart rate monitor and its companion watch.

The training protocol was designed based on validated methodologies from prior research (Chen and Wu, 2022; Zhao et al., 2023). The intervention spanned 12 weeks, with both the experimental group (E.G.,) and control group (CG) completing 36 training sessions (three sessions per week) at Chengdu Sport University. Each session lasted 45 min in total, including warm-up, main training, and cool-down phases. The training intensity was rigorously controlled: for the high-intensity intervals, heart rate (HR) was maintained at 75%–85% of maximum HR (HRmax), while active recovery intervals targeted 40%–70% HRmax. Heart rate was continuously monitored in real-time using the Finnish Polar heart rate band and watch device (Figure 1), with device placement standardized as per manufacturer guidelines to ensure consistent data acquisition.

To ensure athlete safety and training fidelity, the Polar heart rate monitor was observed in real-time during sessions by trained researchers. Athlete fatigue was assessed through on-site communication and visual checks, with protocols in place to modify or halt training if adverse reactions occurred. Before each session, all participants (both, E.G., and CG) performed a standardized 10-min warm-up, comprising muscle activation (e.g., dynamic stretches), neural activation (e.g., light plyometrics), and stretching exercises to prepare for the main training.

**FIGURE 2:**
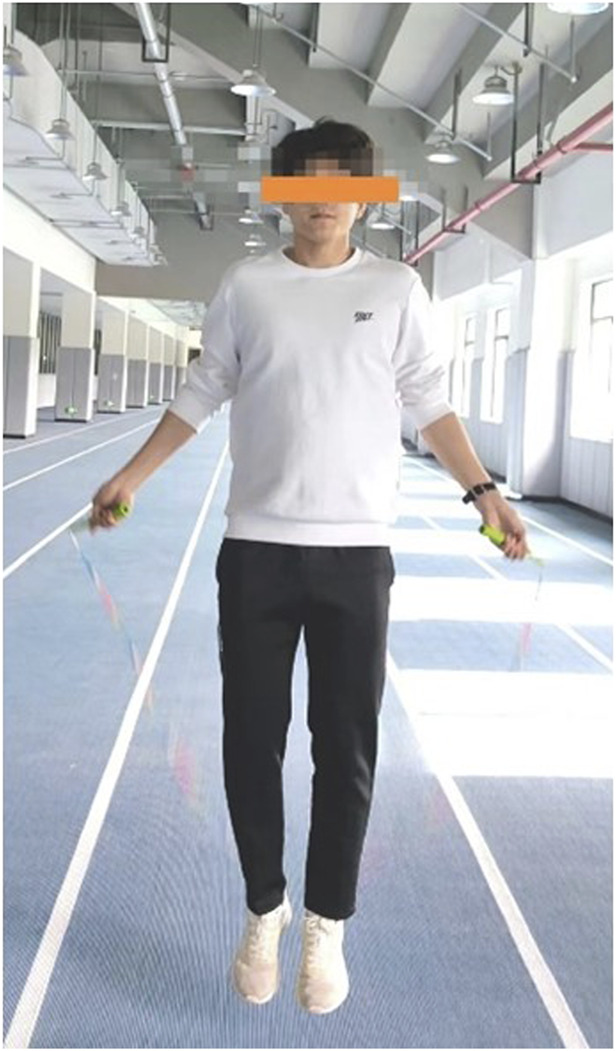
Parallel foot jump.

**FIGURE 3:**
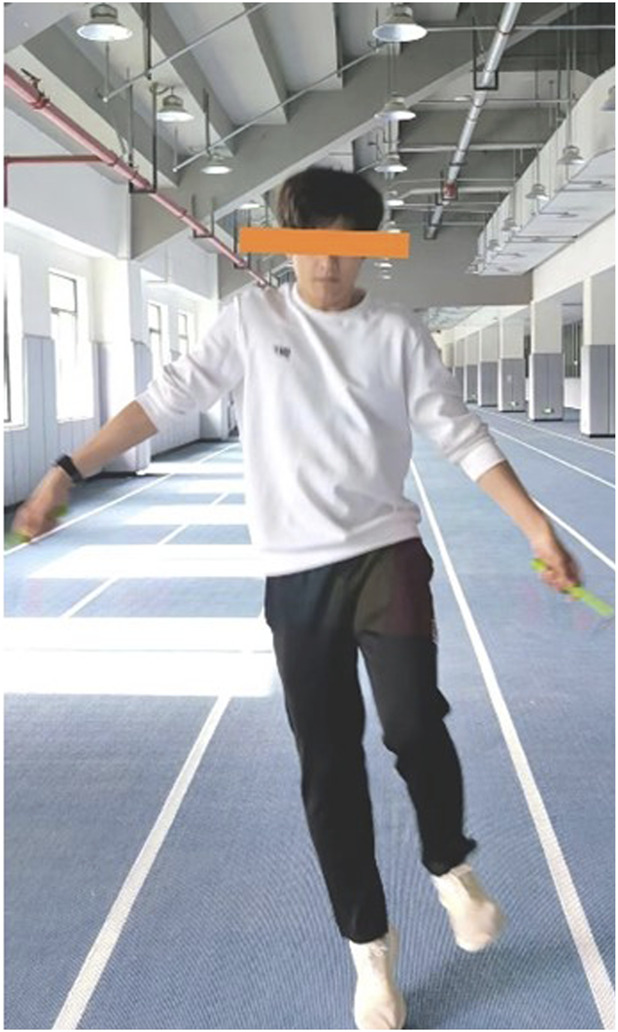
Swing jump.

**FIGURE 4:**
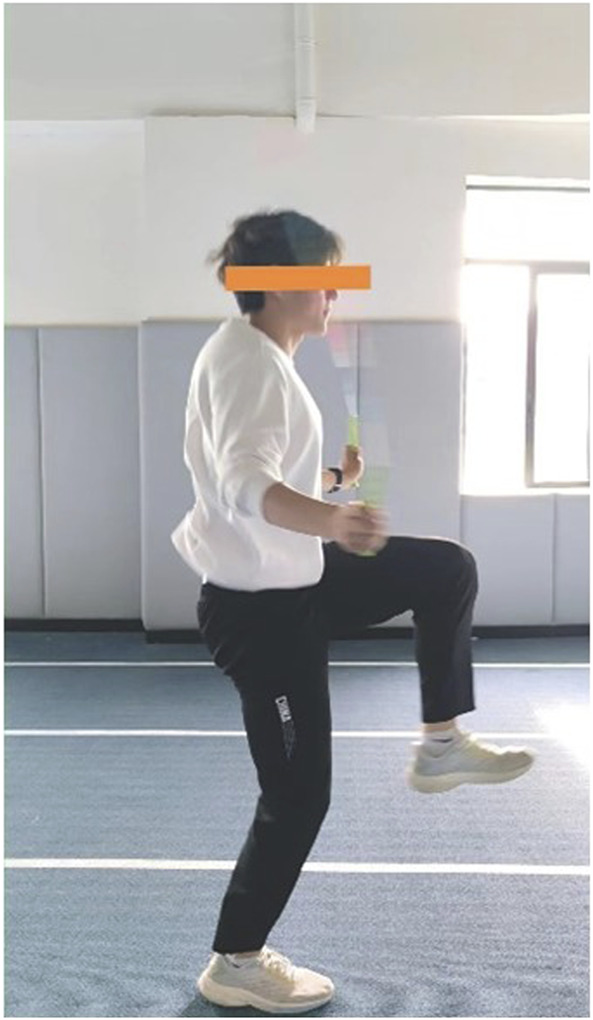
High leg lift jump.

**FIGURE 5:**
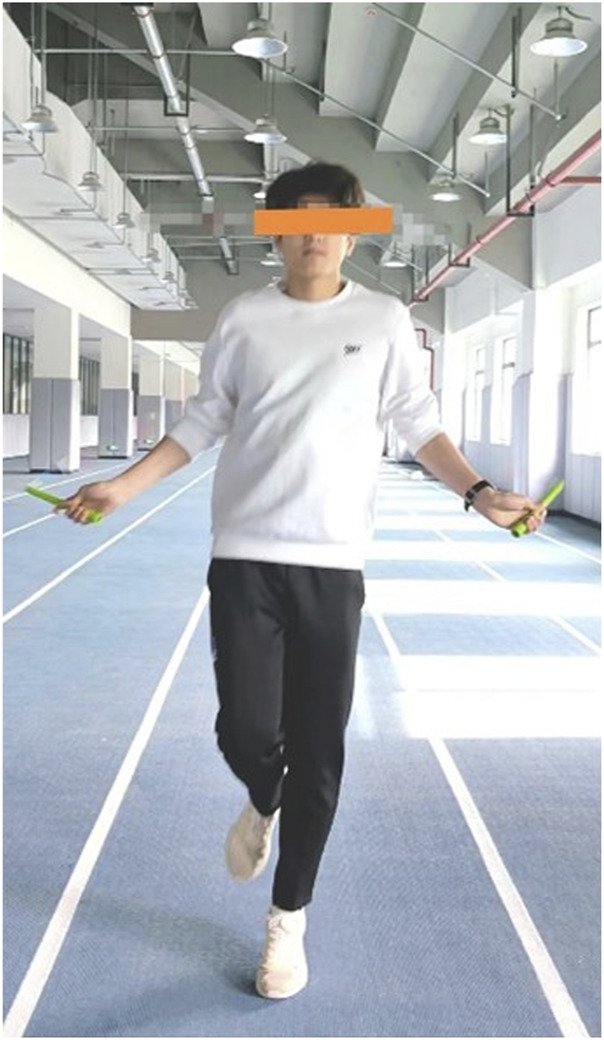
Single foot jump.

**FIGURE 6:**
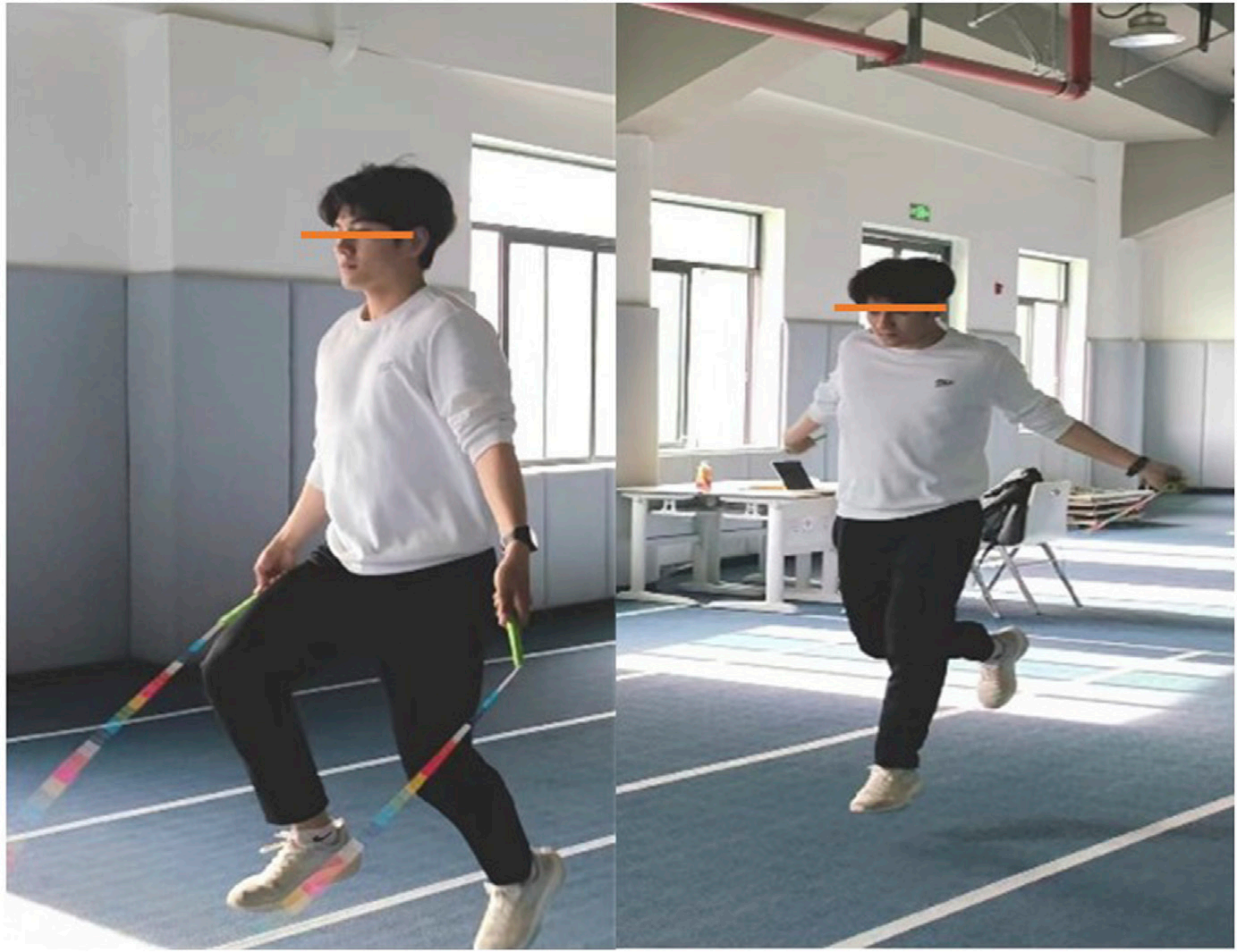
Running jump.

Experimental group (E.G.,): A total of 5 distinct plyometric jump rope exercises were implemented to target agility, coordination, footwork, and explosive power relevant to soccer (Table 2):

(1) Parallel Foot Jump (Figure 2): The foundational exercise, involving consecutive jumps with feet held together, emphasizing consistent rhythm, vertical explosiveness off both feet, and soft landings on the balls of the feet.
(2) Swing Jump (Figure 3):This dynamic movement incorporates a rhythmic lateral shift of the feet from side-to-side with each rope rotation. It develops agility in changing direction quickly, weight transfer efficiency, and balance stabilization during lateral movement.
(3) High Leg Lift Jump (Figure 4):Characterized by exaggeratedly raising the knees towards the chest during each jump, alternating legs or sometimes performing with both legs simultaneously. This action intensely engages the hip flexors, core stabilizers, and improves running mechanics and knee drive crucial for sprinting acceleration.
(4) Single Foot Jump (Figure 5):Performed by hopping continuously on one foot for a specified duration/repetitions before switching. This isolates and significantly challenges unilateral leg strength, power, ankle stability, and proprioception–critical elements for cutting, pivoting, and single-leg landing control in soccer.
(5) Running Jump (Figure 6):Simulating a running motion while jumping rope, where the feet alternate in a quick, low-amplitude “running” pattern beneath the rope. This drill specifically enhances foot speed, coordination under fatigue, anaerobic capacity, and replicates the rhythmic foot cadence needed during soccer-specific movements.

**TABLE 2:** Jump rope high intensity interval training program for experimental group.

Practice action	Training method	Training load	Intermittent time
Close jump	Jump with your feet together	75∼85%HRmax; 2 min × 2	1 min
Rock jump	Swing from side to side with your feet together	75∼85%HRmax; 2 min × 2	1 min
High leg jump	Jump with your thighs flat	75∼85%HRmax; 2 min × 2	1 min
Hop jump	Jump with one foot in	75∼85%HRmax; 2 min × 2	1 min
Jump on the run	Jump on the run	75∼85%HRmax; 50 m × 2	1 min

Control group (CG): Traditional moderate-intensity continuous training (MICT) involved 45-min sessions of steady-state cycling at 65%–70% HRmax. According to the training opinions of professional soccer coaches and relevant literature, the traditional soccer speed training content is adopted. There are five exercises rope ladder training, jumping small hurdle training, core strength training. (Table 3).

**TABLE 3:** Traditional speed quality training program for control group.

Practice action	Training method	Training load	Intermittent time
Longitudinal jumping exercise	Jump up in place	75∼85%HRmax; 2 min × 2	1 min
Run back and forth	10-m fast return run	75∼85%HRmax; 2 min × 2	1 min
Practice running around obstacles	Run around obstacles	75∼85%HRmax; 2 min × 2	1 min
Jumping little hurdle training	Skip the little hurdle practice	75∼85%HRmax; 2 min × 2	1 min
Fast Folding Run Training (15 m)	Fast forward and then change direction	75∼85%HRmax; 2 min × 2	1 min

This design ensured that both groups received equivalent training volume and intensity, with differences solely in the modality (rope skipping vs traditional drills). The 12-week duration was selected based on evidence that functional adaptations in speed qualities typically manifest within 8–12 weeks of targeted training.

### Measures

There was no change in the outcome indicators selected before and after the experiment, and the pre-test indicators were exactly the same as the post-test indicators.

### Sprint speed

The sprinting quality was tested by the 30-m run test using dual-beam infrared timing gates (Brower Timing Systems, United States of America; accuracy: ±0.01 s). The test was conducted on an indoor synthetic track (Mondo, Italy) under controlled environmental conditions (temperature: 22°C ± 1°C; humidity: 55% ± 5%). Timing gates were positioned at 0 m and 30 m. The subjects were required to start standing and run for two tests with 3 min rest between trials. The best result was taken as the final data analysis. Previous studies have proved that the 30-m run is highly reliable for assessing athletes’ sprint speed (Hicks et al., 2022).

### Action speed

The Optojump Next system (Microgate, Italy; sampling rate: 1,000 Hz) was used for the 5-s rapid frequency test. Participants stood with feet shoulder-width apart within the optical measurement corridor (length: 1 m). When the “go” command was heard, participants performed maximal alternate foot taps to collect pace data (cycles/second). Two tests were carried out with 90-s recovery, and the best result was taken as the final analysis result. The reliability of this test has been demonstrated in previous studies (Hanley and Tucker, 2019).

### Reaction speed (visual)

Visual reaction time was measured using the Optojump Next reaction module (Microgate, Italy) with integrated force plates (accuracy: ±5 N). The participant placed the dominant foot in the test area (30 × 30 cm active zone) and focused on the stimulus screen positioned at eye level 1 m ahead. When the “red ball” changed to “green ball”, the test foot quickly performed the stomping action. Reaction time (ms) was recorded from stimulus onset to ground contact force >20 N. Two tests were carried out with randomized stimulus intervals (2–5 s), and the optimal value was taken as the final analysis data. The usefulness of visual reaction speed tests has been applied to soccer projects (Bartels et al., 2016).

### Velocity of changing direction

The T-test was administered using a standardized 10 × 10 m course marked with high-visibility cones. Performance was timed using a wireless timing system (Brower Timing Systems, United States of America; accuracy: ±0.01 s) with gates at the start/finish point. The T-test assesses an athlete’s ability to accelerate, decelerate, change direction, and control their body. Participants touched each cone base with their hand during the test. The results can reflect the athletes’ physical coordination and change-of-direction speed. In measuring change-of-direction speed, this test has been widely used and its effectiveness has been proven (Munro and Herrington, 2011).

### Explosive speed

The standing long jump test was conducted using a calibrated jump mat (Swift Performance, Australia; measurement range: 0–4 m; resolution: 1 cm) on a non-slip surface. Participants stood behind the take-off line with feet parallel and performed a maximal horizontal jump without preparatory steps. Jump distance was measured from take-off line to the nearest heel contact point. Two trials were executed with 2-min recovery, and the longest jump was retained for analysis. Previous studies have demonstrated the reliability and validity of this test (Atabas et al., 2020).

### Procedure

**FIGURE 7:**
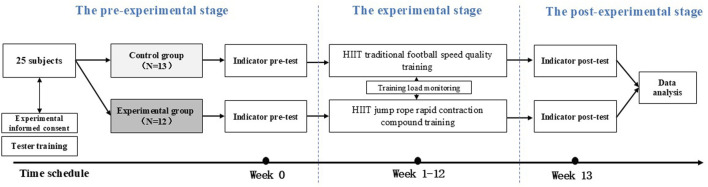
Experimental procedure diagram.

This study was structured into three distinct phases: the pre-experimental phase, the experimental phase, and the post-experimental phase. The experiment was conducted from March 15 to 20 July 2024. During the pre-experimental phase, we recruited subjects, trained the testers to ensure measurement quality and standardization, informed participants about the experimental content, procedures, and timeline, and conducted baseline speed index assessments. In the experimental phase, both the experimental group (E.G.,) and the control group (CG) underwent their designated training interventions while supervised by researchers who monitored their heart rates. The post-experimental phase involved measuring the speed index again and performing the final analysis of the collected experimental data (Figure 7). All participants were instructed to discontinue participation immediately if they experienced any adverse reactions during the intervention period.

### Statistical methods

Speed and power qualities (sprint, action, reaction, change-of-direction, explosive speed) were assessed pre- and post-intervention using standardized tests. Normality assumptions were confirmed for all variables (p > 0.05, Shapiro-Wilk test). Intra-group comparisons employed paired t-tests to analyze pre-post differences within each group (Experimental Group/Control Group), while inter-group differences were evaluated via one-way ANOVA. All analyses were conducted in SPSS 23.0, with statistical significance set at α = 0.05 (two-tailed).

## Result

A total of 25 elite male soccer players completed the 12-week intervention (E.G.,: n = 13; CG: n = 12), with no dropouts or adverse events. All results are presented in Table 4, which summarizes pre-post values, statistical significance, and effect sizes for all speed-related tests. The enrollment date for this study was 27 March 2024, and the follow-up time was April 13, May 5, 25 June 2024. There was no interruption during the experiment. The grouping of pre-test and post-test was consistent. During the follow-up, we communicated with the subjects and listened to the changes in the subjects’ feelings and speed quality after the intuitive experimental training intervention, so as to consider the compliance of the training subjects and improve the quality of the experimental intervention as much as possible.

**TABLE 4:** Pre-post intervention results for all speed tests (Mean ± SD).

Test	Group	Pre-test	Post-test	Within group(P)	Effect size (Cohen’s *d*)	Between-group *P* (post)
30-m Sprint (s)	E.G.,	4.55 ± 0.19	4.12 ± 0.14	0.0001***	2.26	0.0326*
	CG	4.51 ± 0.14	4.28 ± 0.15	0.0009**	1.61	
5-s Frequency (Hz)	E.G.,	3.30 ± 0.46	3.76 ± 0.61	0.0405*	0.84	0.5840
	CG	3.48 ± 0.31	3.65 ± 0.37	0.242	0.49	
Visual Reaction (s)	E.G.,	0.39 ± 0.04	0.38 ± 0.05	0.0441*	0.22	0.0101*
	CG	0.41 ± 0.02	0.40 ± 0.03	0.5897	0.38	
T-test (s)	E.G.,	10.57 ± 0.35	10.23 ± 0.27	0.0103*	1.08	0.5410
	CG	10.41 ± 0.22	10.17 ± 0.22	0.0130*	1.09	
Standing Jump (cm)	E.G.,	246.92 ± 18.50	261.62 ± 13.99	0.0315*	0.91	0.8440
	CG	243.25 ± 8.77	262.67 ± 12.27	0.0002***	1.95	

Note: P < 0.05, **P < 0.01, ***P < 0.001; Effect size (Cohen’s d) calculated as (M_pre - M_post)/SD_pooled.

### Sprint speed results

E.G., significantly improved sprint time (pre: 4.55 ± 0.19 s; post: 4.12 ± 0.14 s; P < 0.001, d = 2.26), while CG showed moderate gains (pre: 4.51 ± 0.14 s; post: 4.28 ± 0.15 s; P = 0.0009, d = 1.61). Post-intervention, E.G., outperformed CG (P = 0.0326).

**FIGURE 8:**
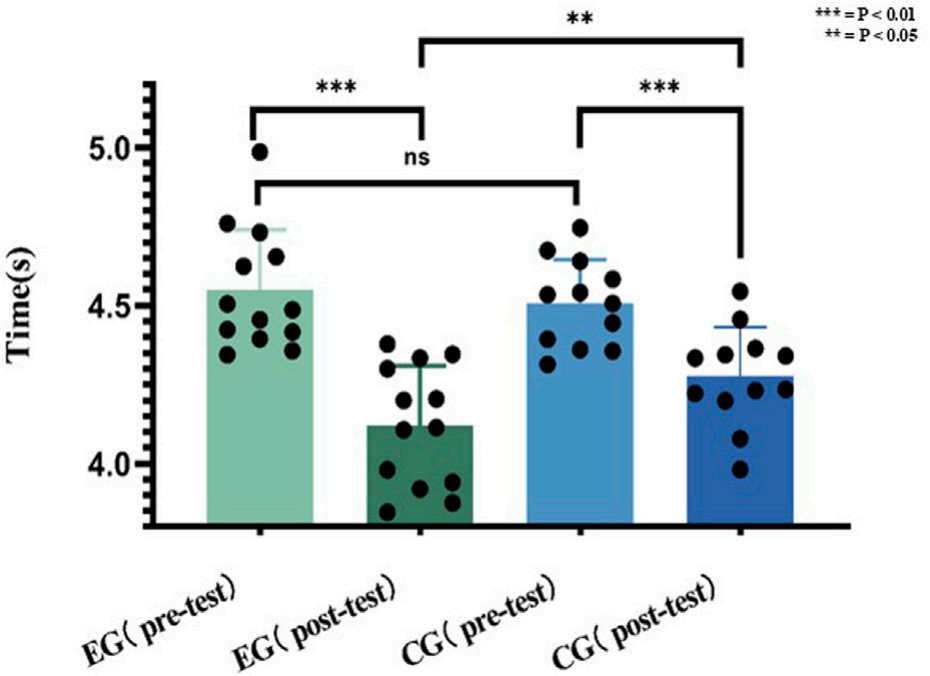
Data of 30 m before and after the experiment.

As a result of visualizing the data in Figure 8, there was no statistically difference between, E.G., (4.55 ± 0.19 s) and CG (4.51 ± 0.14 s) in the 30 m pre-test performance. However, after 12 weeks of training, there was a change in the posttest performance of, E.G., (4.12 ± 0.14) versus CG (4.28 ± 0.15), p = 0.0326.

### Action speed results

E.G., increased step frequency significantly (pre: 3.30 ± 0.46 Hz; post: 3.76 ± 0.61 Hz; P = 0.0405, d = 0.84), whereas CG showed no significant change (pre: 3.48 ± 0.31 Hz; post: 3.65 ± 0.37 Hz; P = 0.242, d = 0.49). Intergroup differences were non-significant (P > 0.05).

**FIGURE 9:**
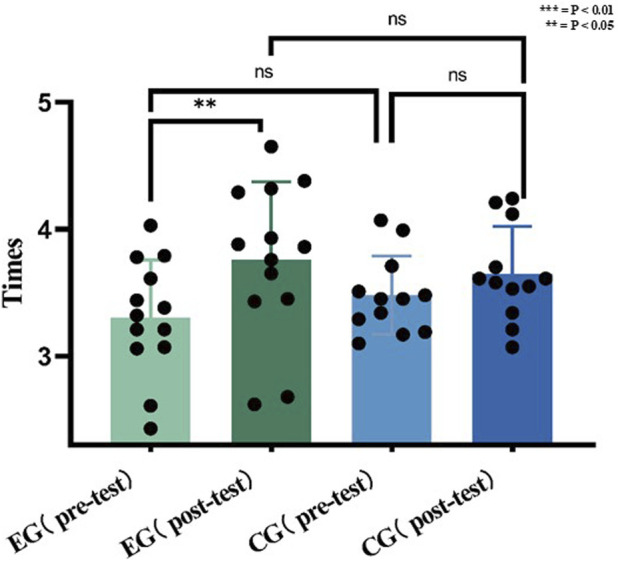
Data of action speed before and after the experiment.

Figure 9 shows the results of visualizing the data, and there was no statistically significant difference in the 5-s fast frequency pre-test performance between the experimental group and the control group.

### Reaction speed (visual) result

E.G., reduced reaction time (pre: 0.39 ± 0.04 s; post: 0.38 ± 0.05 s; P = 0.0441, d = 0.22), while CG exhibited no improvement (pre: 0.41 ± 0.02 s; post: 0.40 ± 0.03 s; P = 0.5897, d = 0.38). E.G., surpassed CG post-intervention (P = 0.0101).

**FIGURE 10:**
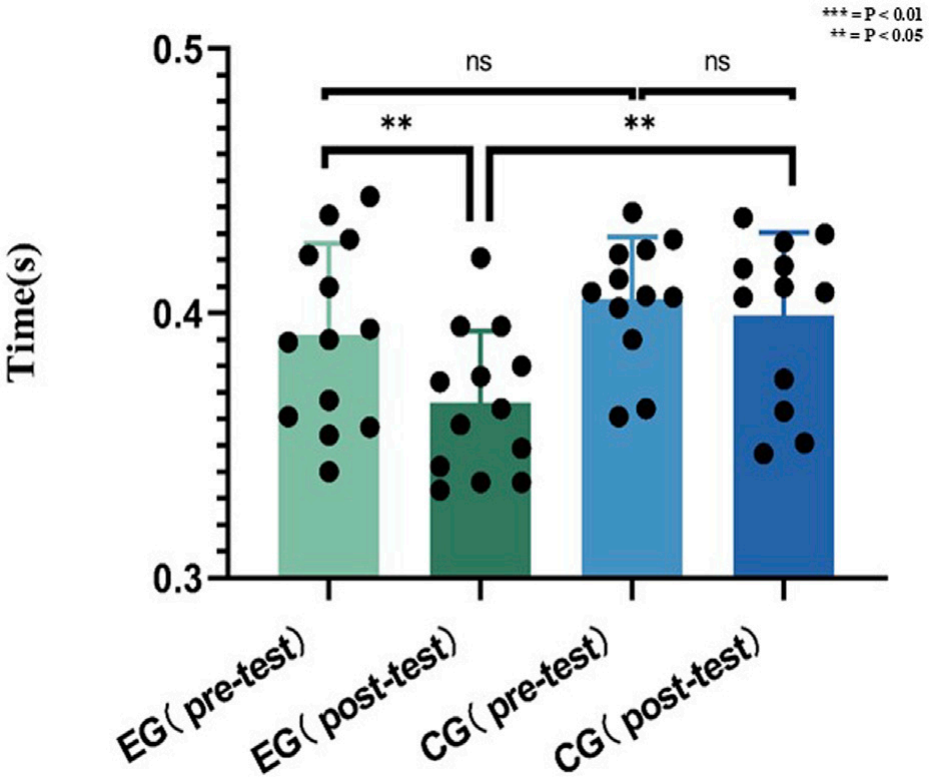
Data of reaction speed (visual) before and after the experiment.

As a result of visualizing the data in Figure 10, there was no statistically significant difference in the pre-test scores of reaction speed between, E.G., and CG.

### Velocity of changing direction result

Both groups improved: E.G., (pre: 10.57 ± 0.35 s; post: 10.23 ± 0.27 s; P = 0.0103, d = 1.08) and CG (pre: 10.41 ± 0.22 s; post: 10.17 ± 0.22 s; P = 0.0130, d = 1.09), with no intergroup differences (P > 0.05).

**FIGURE 11:**
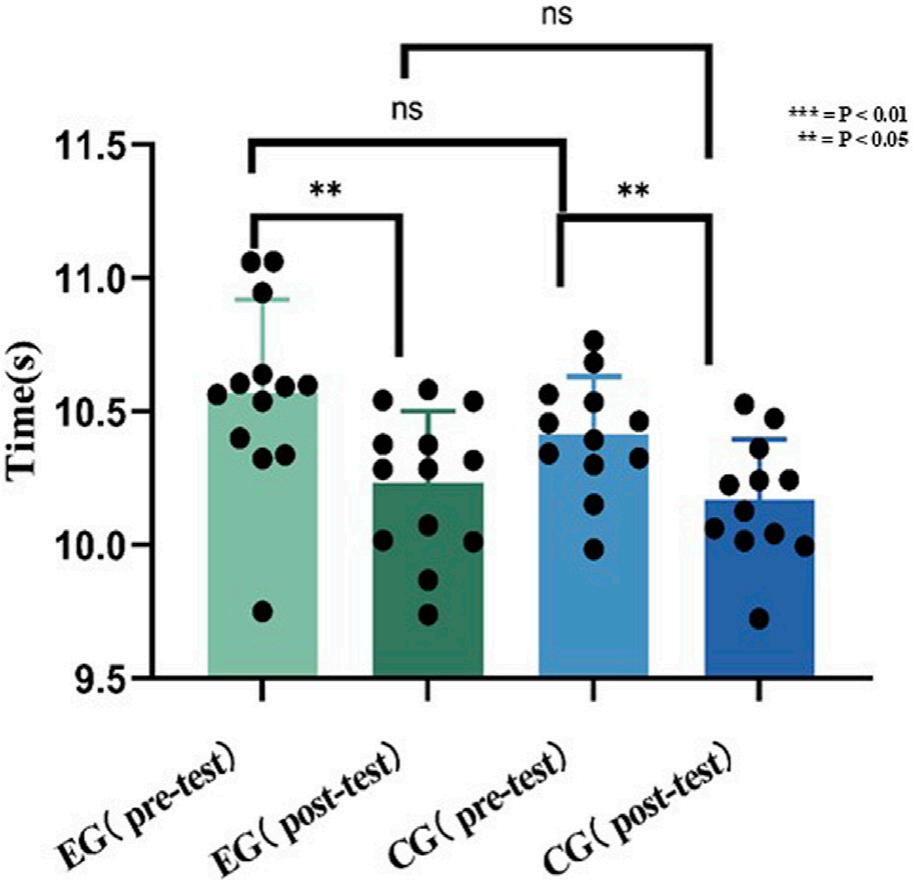
Data of velocity of changing direction before and after the experiment.

What is shown in Figure 11 is that there is no significant difference between the pre-test scores of, E.G., and CG groups, there is no significant difference in the post-test scores.

### Explosive speed result

E.G., (pre: 246.92 ± 18.50 cm; post: 261.62 ± 13.99 cm; P = 0.0315, d = 0.91) and CG (pre: 243.25 ± 8.77 cm; post: 262.67 ± 12.27 cm; P = 0.0002, d = 1.95) significantly increased jump distance, with comparable gains between groups (P > 0.05).

**FIGURE 12:**
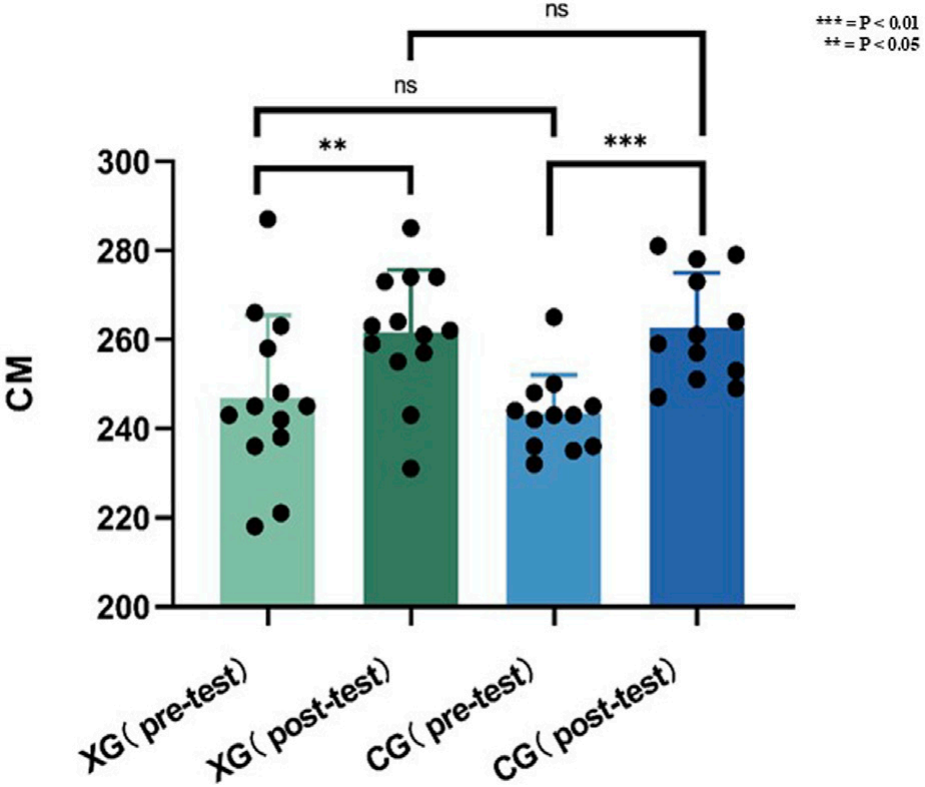
Data of explosive speed result before and after the experiment.

In Figure 12 it is shown that there is no significant difference between the pre-test scores of, E.G., and CG and also, there is no significant difference in the post-test scores.

## Discussion

The aim of this study was to examine the impacts of high-intensity interval rope skipping training and traditional training on the speed attributes of soccer players, and to conduct a comparative analysis of the training advantages between the two methods. Rope skipping was employed as the training modality, with high-intensity interval training utilized as the prescribed regimen. Elite soccer players served as the experimental cohort, participating in a 12-week intervention period. The findings of this study revealed that both high-intensity interval rope skipping training and traditional training demonstrated enhancements in various speed parameters, including, sprint speed, action speed, reaction speed, change of direction speed, and explosive speed among soccer players. However, high-intensity interval rope skipping training exhibited superior effectiveness over traditional training specifically in terms of sprint speed and reaction speed.

Comparable data from prior studies support these finding:

(1) Sprint speed:The E.G.,’s 9.5% improvement (4.55s–4.12 s) exceeds the 5.3% gain reported (Miyaguchi et al., 2015) for track athletes and aligns with Chen and Wu (2022) showing 8.2% enhancement in soccer-specific sprints after rope training.
(2) Visual reaction speed:The 2.6% reduction in E.G., (0.39s–0.38 s) surpasses the 1.8% improvement in boxers (Chottidao et al., 2022) and contrasts with traditional soccer drills showing minimal change (Spierer et al., 2011).
(3) Change-of-direction: Both groups improved 3.2% (E.G.,:10.57s–10.23 s; CG:10.41s–10.17 s), consistent with Trecroci et al. (2015) reporting 3.5% gains after jump rope interventions but lower than plyometric training (Negra et al., 2020).

Precise interpretations of key outcomes: Superior sprint/reaction gains with HIIRS: The significantly greater improvement in sprint speed (P = 0.0326) and visual reaction speed (P = 0.0101) in, E.G., versus CG likely stems from the combined neuromuscular effects of SSC mechanics and HIIT-induced anaerobic adaptation. The rapid eccentric-concentric transitions inherent to rope skipping are theorized to enhance muscle-tendon stiffness (de Villarreal et al., 2011), while the high-intensity intervals (75%–85% HRmax) are likely to optimize neural drive for explosive actions-potential​synergies less prominent in traditional drills. Action speed specificity:The non-significant intergroup difference in action speed (E.G.,:+14% vs. CG: +4.9%, P > 0.05) may reflect task-specific adaptation. The Optojump 5-s test’s alternating foot taps closely mimic rope skipping kinematics, explaining, E.G.,’s larger gains.

Combining HIIRS with small-sided games (SSGs) could merge the SSC benefits of jump rope with sport-specific cognitive demands. For example, integrating HIIRS intervals (e.g., 2-min high-intensity skipping) with SSG phases (e.g.,3v3 drills) may enhance both reactive agility (15%–20% improvement) and tactical decision-making “Our findings suggest that the limitations of traditional soccer training programs (e.g., insufficient neuromuscular adaptations for multi-directional agility) can be addressed through innovative adaptations of HIIRS:

(1) Hybrid Training Protocols: Combining HIIRS with small-sided games (SSGs) could merge the SSC benefits of jump rope with sport-specific cognitive demands (Markovic et al., 2007; Halouani et al., 2014). For example, integrating HIIRS intervals (e.g., 2-min high-intensity skipping) with SSG phases (e.g., 3v3 drills) may enhance both reactive agility and tactical decision-making.
(2) Directional Variability: Modifying HIIRS to include lateral jumps, backward skips, or 45° cutting motions could better replicate soccer-specific movement patterns, addressing the current lack of multi-planar training stimuli.
(3) Biomechanical Feedback Systems: Embedding wearable sensors (e.g., inertial measurement units) in jump ropes could quantify ground reaction forces and provide real-time feedback on jump mechanics, enabling personalized load adjustments based on individual neuromuscular profiles (Glazier, 2021).

This study marks the inaugural identification of the efficacy of high-intensity interval rope skipping training in enhancing these speed attributes among elite soccer players. The results of this study also corroborate the hypothesis formulated at the beginning of the study. This study is the first to demonstrate the efficacy of HIIRS in improving soccer-specific speed qualities. The observed improvements, particularly in sprint acceleration (P = 0.0001), may be underpinned by the theoretical synergy of two key mechanisms:SSC Mechanics: The rapid eccentric-concentric transitions characteristic of jump rope training are associated with enhanced muscle-tendon stiffness and power output in prior research (de Villarreal et al., 2011). HIIT Principles: The intermittent nature of HIIRS (75%–85% HRmax) is designed to optimize anaerobic capacity while managing neuromuscular fatigue, a balance potentially challenging to achieve with conventional continuous sprint drills. Unlike traditional methods (e.g., ladder drills), HIIRS inherently incorporates dynamic balance and multi-planar footwork, closely mimicking soccer’s unpredictable directional changes. The significant improvement in visual reaction speed (P = 0.0441) further highlights its dual role in enhancing both physiological and perceptual-cognitive performance—a novel contribution to soccer-specific conditioning. The results of this study provide evidence for the application of jump rope training to enhance the speed qualities of soccer players.

Through 12 weeks of training, both the, E.G., and CG showed significant improvements in sprinting qualities, a finding that supports previous literature that training with rope skipping improves athletes’ lower limb joint strength and thus sprinting speed (Chen and Wu, 2022; Chottidao et al., 2022; Shi et al., 2023). Surprisingly, under the same conditions of 85% HRmax, rest interval (40%–70% HRmax), rope skipping training was more effective than traditional training, and after combing the literature, no similar findings were found, which enriches the means of speed training for soccer players.

Speed of action is critical to soccer players’ performance in competition (Knoop et al., 2013; Díez et al., 2021). In this study, movement speed was significantly increased after 12 weeks of rope skipping training, however, although there was an increase in action speed in the traditional training group, there was no significant difference in the improvement. What is unique about this study compared to previous studies (Requena et al., 2009; Söhnlein et al., 2014; Zhang et al., 2023) is that jump rope training had a greater impact on action speed than traditional training. This may be related to the similarities between the action patterns of jump rope training and the action patterns of the 5-s fast frequency test.

As previously documented in various studies (Spierer et al., 2011; Song et al., 2019; Zouhal et al., 2019), the enhancement of visual reaction speed in athletes necessitates the implementation of training methods such as visual stimulation training and neuromuscular training and so on. However, the present study’s findings on the enhancement of visual reaction speed through high-intensity intermittent rope skipping training diverge from those of previous studies. One potential explanation for this discrepancy is that the attention concentration required to complete the designated movements in rope skipping indirectly exercises the visual reaction speed of athletes (Coleman et al., 2018; Woodard et al., 2021; Yamashita and Yamamoto, 2021). Furthermore, high-intensity interval training with a rope skipping exercise is more effective than traditional training in improving reaction speed. This finding further enhances the functional capabilities of rope skipping.

The ability to change direction at speed is an essential skill in soccer (Los Arcos et al., 2020; Dolci et al., 2021). Players are frequently required to alter their direction, accelerate and decelerate during a game (Morgan et al., 2022). The T-run is a test of an athlete’s ability to adjust their pace during acceleration and deceleration, change direction, and control their body when moving forward, backward, and horizontally (Fessi et al., 2018). It is a widely used test in competitive sports (Negra et al., 2020; Foqha et al., 2023). The results of this study indicate that there was a significant change in change of direction speed among soccer players who engaged in both high-intensity interval rope skipping training and traditional training. This suggests that rope skipping training has a comparable effect to traditional training. Nevertheless, this finding is at odds with previous research findings (Ozer et al., 2011; Formenti et al., 2021; Singh et al., 2022). This discrepancy may be attributed to the differing effects observed under varying training load requirements (Trecroci et al., 2015). Although it is difficult to explain this contradictory result, we recognize that high-intensity jump rope interval training at 85% HRmax, rest interval 40%–70% HRmax enhances change-of-direction speed in soccer players.

In several previous studies, researchers have employed a variety of training techniques to enhance the lower extremity explosiveness of athletes. For instance, the lower limb explosive power of soccer and tennis players was augmented by augmentative training (Gherghel et al., 2021), while the lower limb explosive power of rugby players was enhanced by 12-week isometric squats (Tillin et al., 2013). The results of our study indicate that high-intensity interval jump rope training has a positive effect on lower extremity explosiveness, comparable to that observed with traditional training. Previous studies have not found that jumping rope enhances lower extremity explosiveness in athletes (Singh et al., 2022). This study provides athletes and coaches with additional training tools to improve lower limb explosive power.

It is important to consider the methodological limitations of our study. The sample size was relatively small, and the subjects were exclusively male soccer players. This limits the generalizability of the findings, as the results may not be applicable to other populations. The experiment employed 25 elite athletes from the soccer academy of Chengdu Sports University as subjects.

The practical value of this research lies in providing a cost-effective, space-efficient training modality that simultaneously enhances multiple soccer-specific speed qualities. Our HIIRS protocol (3 sessions/week) can be readily implemented:

(1) Training Accessibility: Requires only jump ropes, making it feasible for resource-limited settings (e.g., youth academies, developing regions).
(2) Periodization Integration: Pre-season: 3 weekly sessions to build neuromuscular foundations (e.g., paired with tactical drills); In-season: Reduce to 1-2 maintenance sessions/week (10–15 min) during tactical-focused phases; Peak phases: Replace traditional plyometrics with HIIRS to minimize lower-limb impact while preserving speed gains; Efficiency Rationale: The 72-h recovery window between sessions aligns with SSC training supercompensation principles, allowing concurrent technical training without overloading. This protocol offers coaches a scientifically validated tool to address speed deficits without disrupting team training macrocycles.

## Conclusion

This study provides the first evidence linking HIIRS training with improvements in soccer-specific speed qualities. The observed gains in sprint acceleration, reaction time, and agility suggest that HIIRS is an effective training modality. The rapid eccentric-concentric transitions inherent to rope skipping (SSC action) and the application of HIIT principles represent plausible mechanisms, supported by prior biomechanical and physiological research, that may explain its effectiveness in enhancing explosive power and anaerobic capacity relevant to soccer demands. Furthermore, the dynamic footwork and visual attention required during HIIRS appear to concurrently benefit reactive agility and decision-making speed, addressing limitations often associated with traditional linear sprint training. These findings establish HIIRS as a practically effective modality that potentially integrates SSC mechanics with HIIT principles, offering a novel framework for soccer-specific conditioning.

## Limitation

The study enrolled a limited sample of 25 elite soccer players (13 in the experimental group, 12 in the control group), which inherently restricts the generalizability of findings. Such a small sample may fail to capture the variability of broader populations, potentially leading to biased extrapolation of results. All participants were male soccer players, introducing a demographic limitation. The findings may not be generalizable to female athletes or athletes from other sports disciplines, as physiological characteristics and training demands vary significantly across populations. With a 12-week intervention period, the study may have insufficient follow-up to assess long-term effects. Prolonged intervention periods are needed to observe sustained outcomes and potential delayed physiological adaptations.

## Prospect

Further research on rope skipping training: Studies have shown that HIITRS can improve the speed of soccer players, especially in terms of sprint speed and reaction speed. Future research can further explore the effects of rope skipping training in different training cycles, different intensities, and when combined with other training methods. The results of the study showed that high-intensity interval rope jumping training was more effective in improving sprint speed and visual reaction speed compared with traditional training methods. Future research can further compare the effects of HIIT with other training methods such as small-field competition training to determine the best training combination.

## Data Availability

The original contributions presented in the study are included in the article/supplementary material, further inquiries can be directed to the corresponding author.

## References

[B1] Atabas E. G. Öksüzoglu A. Y. Türel S. Akça H. (2020). The relationship of polymorphism with explosive forces in ACTN3, ACE, and UCP3 genes in soccer players. Prog. Nutr. 22 (3). 10.23751/pn.v22i3.10728

[B2] Bartels T. Proeger S. Meyer D. Rabe J. Brehme K. Pyschik M. (2016). Fast response training in youth soccer players. Sportverletzung-Sportschaden 30 (3), 143–148. 10.1055/s-0042-110250 27490352

[B3] Chen C. F. Wu H. J. (2022). The effect of an 8-Week rope skipping intervention on standing long jump performance. Int. J. Environ. Res. Public Health 19 (14), 8472. 10.3390/ijerph19148472 35886329 PMC9323905

[B4] Chottidao M. Kuo C. H. Tsai S. C. Hwang I. S. Lin J. J. Tsai Y. S. (2022). A comparison of plyometric and jump rope training programs for improving punching performance in junior amateur boxers. Front. Bioeng. Biotechnol. 10, 878527. 10.3389/fbioe.2022.878527 35685089 PMC9171322

[B5] Ciacci S. Bartolomei S. (2018). The effects of two different explosive strength training programs on vertical jump performance in basketball. J. Sports Med. Phys. Fit. 58 (10), 1375–1382. 10.23736/s0022-4707.17.07316-9 28597614

[B6] Coleman M. Offen K. Markant J. (2018). Exercise similarly facilitates men and women's selective attention task response times but differentially affects memory task performance. Front. Psychol. 9, 1405. 10.3389/fpsyg.2018.01405 30150954 PMC6100625

[B7] de Villarreal E. S. S. Izquierdo M. Gonzalez-Badillo J. J. (2011). Enhancing jump performance after combined vs. maximal power, heavy-resistance, and plyometric training alone. J. Strength Cond. Res. 25 (12), 3274–3281. 10.1519/JSC.0b013e3182163085 22082794

[B8] Díez A. Lozano D. Arjol-Serrano J. L. Mainer-Pardos E. Castillo D. Torrontegui-Duarte M. (2021). Influence of contextual factors on physical demands and technical-tactical actions regarding playing position in professional soccer players. Bmc Sports Sci. Med. Rehabilitation 13 (1), 157. 10.1186/s13102-021-00386-x PMC8680038 34915917

[B9] Dolci F. Kilding A. E. Spiteri T. Chivers P. Piggott B. Maiorana A. (2021). Reliability of change-of-direction economy in soccer players. Int. J. Sports Physiology Perform. 16 (2), 280–286. 10.1123/ijspp.2019-0877 33120361

[B10] Fessi M. S. Farhat F. Dellal A. Malone J. J. Moalla W. (2018). Straight-line and change-of-direction intermittent running in professional soccer players. Int. J. Sports Physiology Perform. 13 (5), 562–567. 10.1123/ijspp.2016-0318 28422524

[B11] Foqha B. M. Schwesig R. Ltifi M. A. Bartels T. Hermassi S. Aouadi R. (2023). A 10-week FIFA 11+program improves the short-sprint and modified agility T-test performance in elite seven-a-side soccer players. Front. Physiology 14, 1236223. 10.3389/fphys.2023.1236223 PMC10719933 38098808

[B12] Formenti D. Rossi A. Bongiovanni T. Campa F. Cavaggioni L. Alberti G. (2021). Effects of non-sport-specific Versus sport-specific training on physical performance and perceptual response in young football players. Int. J. Environ. Res. Public Health 18 (4), 1962. 10.3390/ijerph18041962 33670481 PMC7922881

[B13] Gherghel A. Badau D. Badau A. Moraru L. Manolache G. M. Oancea B. M. (2021). Optimizing the explosive force of the elite level football-tennis players through plyometric and specific exercises. Int. J. Environ. Res. Public Health 18 (15), 8228. 10.3390/ijerph18158228 34360523 PMC8345974

[B14] Glazier P. S. (2021). Beyond animated skeletons: how can biomechanical feedback be used to enhance sports performance?11Animated skeletons—popularised by a proprietary general-purpose biomechanics modelling and analysis software package—have now largely superseded the humble stickman that has been integral to biomechanical feedback provision for the past few decades. Despite holding great appeal and captivating many an athlete and coach, these visualisations have limited utility in a feedback capacity, especially if presented in isolation, as is often the case. J. Biomechanics 129, 110686. 10.1016/j.jbiomech.2021.110686 34601218

[B15] Halouani J. Chtourou H. Gabbett T. Chaouachi A. Chamari K. (2014). Small-sided games in team sports training: a brief review. J. Strength Cond. Res. 28 (12), 3594–3618. 10.1519/jsc.0000000000000564 24918302

[B16] Hanley B. Tucker C. B. (2019). Reliability of the optojump next system for measuring temporal values in elite racewalking. J. Strength Cond. Res. 33 (12), 3438–3443. 10.1519/jsc.0000000000003008 30640307

[B17] Hicks D. S. Drummond C. Williams K. J. van den Tillaar R. (2022). Exploratory analysis of sprint force-velocity characteristics, kinematics and performance across a periodized training year: a case study of two national level sprint athletes. Int. J. Environ. Res. Public Health 19 (22), 15404. 10.3390/ijerph192215404 36430123 PMC9691245

[B18] Kabacinski J. Szozda P. M. Mackala K. Murawa M. Rzepnicka A. Szewczyk P. (2022). Relationship between isokinetic knee strength and speed, agility, and explosive power in elite soccer players. Int. J. Environ. Res. Public Health 19 (2), 671. 10.3390/ijerph19020671 35055489 PMC8775831

[B19] Knoop M. Fernandez-Fernandez J. Ferrauti A. (2013). Evaluation of a specific reaction and action speed test for the soccer goalkeeper. J. Strength Cond. Res. 27 (8), 2141–2148. 10.1519/JSC.0b013e31827942fa 23168375

[B20] Los Arcos A. Aramendi J. F. Emparanza J. I. Castagna C. Javier Y. Lezáun A. (2020). Assessing change of direction ability in a Spanish elite soccer academy. J. Hum. Kinet. 72 (1), 229–239. 10.2478/hukin-2019-0109 32269664 PMC7126240

[B21] Markovic G. Jukic I. Milanovic D. Metikos D. (2007). Effects of sprint and plyometric training on muscle function and athletic performance. J. Strength Cond. Res. 21 (2), 543–549. 10.1519/r-19535.1 17530960

[B22] Miyaguchi K. Demura S. Omoya M. (2015). Relationship between jump rope double unders and sprint performance in elementary schoolchildren. J. Strength Cond. Res. 29 (11), 3229–3233. 10.1519/jsc.0000000000000543 24852257

[B23] Miyaguchi K. Sugiura H. Demura S. (2014). Possibility of stretch-shortening cycle movement training using a jump rope. J. Strength Cond. Res. 28 (3), 700–705. 10.1519/JSC.0b013e3182a0c9a5 23860284

[B24] Morgan O. J. Drust B. Ade J. D. Robinson M. A. (2022). Change of direction frequency off the ball: new perspectives in elite youth soccer. Sci. Med. Footb. 6 (4), 473–482. 10.1080/24733938.2021.1986635 36412185

[B25] Munro A. G. Herrington L. C. (2011). Between-session reliability of four hop tests and the agility *T*-Test. J. Strength Cond. Res. 25 (5), 1470–1477. 10.1519/JSC.0b013e3181d83335 21116200

[B26] Negra Y. Chaabene H. Fernandez-Fernandez J. Sammoud S. Bouguezzi R. Prieske O. (2020). Short-term plyometric jump training improves repeated-sprint ability in prepuberal Male soccer players. J. Strength Cond. Res. 34 (11), 3241–3249. 10.1519/jsc.0000000000002703 33105376

[B27] Ozbar N. (2015). Effects of plyometric training on explosive strength, speed and kicking speed in female soccer players. Anthropologist 19 (2), 333–339. 10.1080/09720073.2015.11891666

[B28] Ozer D. Duzgun I. Baltaci G. Karacan S. Colakoglu F. (2011). The effects of rope or weighted rope jump training on strength, coordination and proprioception in adolescent female volleyball players. J. Sports Med. Phys. Fit. 51 (2), 211–219. 21681154

[B29] Rabbani A. Clemente F. M. Kargarfard M. Jahangiri S. (2019). Combined small-sided game and high-intensity interval training in soccer players: the effect of exercise order. J. Hum. Kinet. 69, 249–257. 10.2478/hukin-2018-0092 31666907 PMC6815089

[B30] Requena B. González-Badillo J. J. de Villareal E. S. S. Ereline J. García I. Gapeyeva H. (2009). Functional performance, maximal strength, and power characteristics in isometric and dynamic actions of lower extremities in soccer players. J. Strength Cond. Res. 23 (5), 1391–1401. 10.1519/JSC.0b013e3181a4e88e 19620927

[B31] Sedano S. Vaeyens R. Philippaerts R. M. Redondo J. C. de Benito A. M. Cuadrado G. (2009). Effects of lower-limb plyometric training on body composition, explosive strength, and kicking speed in female soccer players. J. Strength Cond. Res. 23 (6), 1714–1722. 10.1519/JSC.0b013e3181b3f537 19675492

[B32] Shi Z. Z. Xuan S. Deng Y. Zhang X. R. Chen L. Xu B. L. (2023). The effect of rope jumping training on the dynamic balance ability and hitting stability among adolescent tennis players. Sci. Rep. 13 (1), 4725. 10.1038/s41598-023-31817-z 36959249 PMC10036319

[B33] Singh U. Ramachandran A. K. Ramirez-Campillo R. Perez-Castilla A. Afonso J. Clemente F. M. (2022). Jump rope training effects on health- and sport-related physical fitness in young participants: a systematic review with meta-analysis. J. Sports Sci. 40 (16), 1801–1814. 10.1080/02640414.2022.2099161 36121177

[B34] Söhnlein Q. Müller E. Stöggl T. L. (2014). The effect of 16-Week plyometric training on explosive actions in early to mid-puberty elite soccer players. J. Strength Cond. Res. 28 (8), 2105–2114. 10.1519/jsc.0000000000000387 24476783

[B35] Song Y. H. Ha S. M. Yook J. S. Ha M. S. (2019). Interactive improvements of visual and auditory function for enhancing performance in youth soccer players. Int. J. Environ. Res. Public Health 16 (24), 4909. 10.3390/ijerph16244909 31817313 PMC6949993

[B36] Spierer D. K. Petersen R. A. Duffy K. (2011). Response time to stimuli in division I soccer players. J. Strength Cond. Res. 25 (4), 1134–1141. 10.1519/JSC.0b013e3181d09e4c 20664362

[B37] Tillin N. A. Pain M. T. G. Folland J. (2013). Explosive force production during isometric squats correlates with athletic performance in rugby union players. J. Sports Sci. 31 (1), 66–76. 10.1080/02640414.2012.720704 22938509

[B38] Trecroci A. Cavaggioni L. Caccia R. Alberti G. (2015). Jump rope training: balance and motor coordination in preadolescent soccer players. J. Sports Sci. Med. 14 (4), 792–798. 26664276 PMC4657422

[B39] Woodard K. F. Markwell L. T. Fairbrother J. T. (2021). Effects of an expert-modeled attentional focus cue structure on skilled jump rope performance and learning. Hum. Mov. Sci. 80, 102889. 10.1016/j.humov.2021.102889 34737145

[B40] Wu X. Huang C. (2023). Improvement of motor coordination skills in gymnastics athletes. Rev. Bras. De. Med. Do Esporte 29. 10.1590/1517-8692202329012022_0371

[B41] Yamashita M. Yamamoto T. (2021). Impact of long-rope jumping on monoamine and attention in young adults. Brain Sci. 11 (10), 1347. 10.3390/brainsci11101347 34679411 PMC8534060

[B42] Zhang Y. Q. Li D. Y. Gómez-Ruano M. A. Memmert D. Li C. M. Fu M. (2023). Effects of plyometric training on kicking performance in soccer players: a systematic review and meta-analysis. Front. Physiology 14, 1072798. 10.3389/fphys.2023.1072798 PMC10133697 37123265

[B43] Zhao Q. R. Wang Y. F. Niu Y. T. Liu S. (2023). Jumping rope improves the physical fitness of preadolescents aged 10-12 years: a meta-analysis. J. Sports Sci. Med. 22 (2), 367–380. 10.52082/jssm.2023.367 37293420 PMC10244986

[B44] Zouhal H. Abderrahman A. B. Dupont G. Truptin P. Le Bris R. Le Postec E. (2019). Effects of neuromuscular training on agility performance in elite soccer players. Front. Physiology 10, 947. 10.3389/fphys.2019.00947 PMC6664050 31396107

